# Evaluation of *Sapindus mukorossi* Gaertn Flower Water Extract on In Vitro Anti-Acne Activity

**DOI:** 10.3390/cimb47050316

**Published:** 2025-04-28

**Authors:** Zibing Zhao, Aohuan Zhang, Liya Song, Congfen He, Huaming He

**Affiliations:** Beijing Key Laboratory of Plant Resources Research and Development, School of Light Industry Science and Engineering, Beijing Technology and Business University, Beijing 100048, China; zzb063005@163.com (Z.Z.); zah18674735264@163.com (A.Z.); congfenhe@126.com (C.H.)

**Keywords:** *Sapindus mukorossi* flower, anti-acne, saponin, antioxidant, anti-inflammation, transcriptome analysis

## Abstract

**Background:** *Sapindus mukorossi* Gaertn is a deciduous tree with saponins as the main active ingredients and has been utilized in medicine and cosmetic industries. Currently, the investigations of *S. mukorossi* mainly focus on the pericarp and seed kernel parts, while other parts are yet to be studied and developed. This study aimed to investigate the anti-acne potential of *S. mukorossi* flower water extract (SMFW) by in vitro experiments. **Methods and Results:** The DPPH, ABTS, superoxide anion radical scavenging assay, and FRAP assay revealed the strong antioxidant activities of SMFW. The antibacterial activity of SMFW against *Cutibacterium acnes* has been evaluated with an inhibition diameter of 14.08 ± 0.63 mm. Furthermore, SMFW significantly inhibited the secretion of pro-inflammatory cytokines (TNF-α, IL-6, and IL-β) in lipopolysaccharide (LPS)-stimulated THP-1 macrophages. Transcriptome analysis showed that SMFW treatment reversed 448 LPS-upregulated DEGs and 349 LPS-downregulated DEGs, and KEGG enrichment analysis indicated that SMFW might exert its anti-inflammatory effect via NOD-like receptor and JAK-STAT signaling pathways. **Conclusions:** SMFW exhibited antioxidant, antibacterial, and anti-inflammatory properties in in vitro experiments. RNA-seq analysis indicated that SMFW may alleviate inflammation by regulating the NOD-like receptor and JAK-STAT signaling pathways. In summary, SMFW has shown potential for anti-acne efficacy and can be used as a natural raw material in cosmetics.

## 1. Introduction

*Sapindus mukorossi* Gaertn (*S. mukorossi*), commonly known the soapnut, belongs to the Sapindaceae family and is widely cultivated in China, Japan, Korea, and India [1]. The fruits, leaves, and roots of *S. mukorossi* have been utilized in traditional medicine to treat eczema, psoriasis, migraine, epilepsy, arthritis, rheumatism, etc. [2]. Saponins, as the main active ingredient of *S. mukorossi*, have been extensively studied due to their strong foaming and cleaning properties, and are widely used as surfactants in food, cosmetic, and pharmaceutical industries [3]. In addition, *S. mukorossi* extract also contains many other bioactive compounds such as flavonoids [4] and polysaccharide [5]. Previous studies have shown that *S. mukorossi* extracts have a variety of bioactivities such as skin whitening, antibacterial [6], anti-inflammation [7], wound healing promotion [8], and anticancer [5].

Currently, research on *S. mukorossi* mainly focuses on the pericarp and seed kernel, while studies on flowers are relatively scarce. Four novel saponins were isolated from alcohol extract of *S. mukorossi* flower, and their neuritogenic activities were evaluated in PC12 cells, suggesting their potential for Alzheimer’s disease treatment [9]. Overall, the biological activities of *S. mukorossi* flower extract remains to be explored.

Acne vulgaris (acne) is a chronic inflammatory condition that affects the sebaceous glands of the skin, which can lead to the occurrence of comedones, pimples, and pustules [10]. Four factors contribute to the pathogenesis of acne, including follicular hyperplasia of sebaceous glands, increased sebum secretion, the overgrowth of *Cutibacterium acnes* (*C. acnes*), and associated inflammatory responses [10]. *C. acnes* can induce the production of inflammatory cytokine in macrophages through entities like Toll-like receptors (TLRs) and NOD-like receptors, and diversified signaling pathways [11]. Specifically, *C. acnes* modulates MAPK and NF-κB signaling pathways via the TLR2 receptor to activate the production of nitric oxide (NO) and prostaglandin E2 (PGE2) [12]. *C. acnes* also engages additional pathways, including the JAK-STAT pathway, thereby modulating the inflammatory response [13]. Moreover, the serum of acne patients exhibited significantly higher levels of malondialdehyde and higher activity of xanthine oxidase (XO), but lower activities of antioxidant enzymes (superoxide dismutase and catalase), indicating that the oxidative damage may contribute to the pathogenesis of acne [14]. Hence, the evaluation of inhibitory activity on *C. acnes* as well as antioxidant and anti-inflammatory potential reflects the anti-acne capacity of natural products. Traditional chemical medications for acne treatment have potential side effects such as skin dryness, redness, and irritation. Therefore, development of new natural anti-acne ingredients with fewer side effects is needed. *S. mukorossi* fruit extract has been demonstrated to possess significant antibacterial properties, thus rendering it a promising candidate for use as a natural antimicrobial agent [6,15]. This botanical extract exhibits multiple therapeutic advantages for acne treatment, including its gentle cutaneous compatibility, potent broad-spectrum antimicrobial activity, high material accessibility, and low cost compared to counterparts.

The present study aimed to evaluate the bioactivities of SMFW for its anti-acne application in pharmaceutical and cosmetic industry. Saponin yields of *S. mukorossi* flower were examined using water as a solvent under different extraction conditions. Then, the in vitro antioxidant and antibacterial activities of SMFW and *S. mukorossi* pericarp water extract (SMPW) were examined. Furthermore, the anti-inflammatory effects of SMFW on LPS-induced THP-1 macrophages were investigated and the underlying mechanism was explored based on RNA-sequencing analysis.

## 2. Materials and Methods

### 2.1. Materials and Chemicals

#### 2.1.1. Plant Materials

*S. mukorossi* flowers were collected in June 2022 and *S. mukorossi* fruits were collected in October 2022 from Sanming city (Fujian province, China). Voucher specimens were confirmed and deposited at the Department of Cosmetics, School of Light Industry Science and Engineering, Beijing Technology and Business University, China. Flowers and pericarps were dried by a vacuum freeze dryer (LGJ-18, Beijing Songyuanhuaxing Technology Develop Co., Ltd. Beijing, China ) and grinded in a grinder. The obtained powders were filtered through a 50-mesh sieve and stored at −20 °C until further processing.

In this study, *S. mukorossi* flower powder was extracted by heating in a water bath, and the specific extraction conditions were as follows: a solid:liquid ratio of 1:30 g:mL, an extraction time of 180 min, and an extraction temperature of 80 °C.

#### 2.1.2. Cell Culture

The human monocytic leukemia cell line (THP-1) was obtained from China National Infrastructure of Cell Line Resource. Cells were cultured in RPMI 1640 medium supplemented with 10% fetal bovine serum and 1% penicillin and streptomycin and maintained in an incubator (37 °C and 5% CO_2_). HaCaT cell line was obtained from China National Infrastructure of Cell Line Resource. Cells were cultured in DMEM medium supplemented with 10% fetal bovine serum and 1% penicillin and streptomycin and maintained in an incubator (37 °C and 5% CO_2_).

#### 2.1.3. Chemicals

Oleanolic acid, rutin, and gallic acid were purchased from Yuanye (Shanghai, China); 2,4,6-Tri(2-pyridyl)-s-triazine (TPTZ) and 1,1-diphenyl-2-picryl-hydrazyl radical were obtained from Macklin (Shanghai, China); 2,2′-Azino-bis(3-ethylbenzothiazoline -6-sulfonic) (ABTS) and sodium dodecyl sulfate (SDS) were purchased from Aladdin (Shanghai, China); defibrinated sheep blood was purchased from Solarbio (Beijing, China); phorbol 12-myristate 13-acetate (PMA) was purchased from AbMole (Shanghai, China); fetal bovine serum and RPMI 1640 medium were purchased from Gibco (New York, NY, USA); CCK-8 kit was purchased from Beyotime (Shanghai, China); dexamethasone was purchased from Yuanye (Shanghai, China); Human Tumor Necrosis Factor α (TNF-α) ELISA kit, Human Interleukin 1β (IL-1β) ELISA kit, and Human Interleukin 6 (IL-6) ELISA kit were purchased from Cusabio (Wuhan, China).

### 2.2. Phytochemical Measurements

#### 2.2.1. Determination of Saponin, Total Flavonoid, and Total Phenol Contents

Determination of saponin contents in the samples was performed according to the vanillin-glacial acetic acid method [16]. The sodium nitrite-aluminium nitrite method [17] was used to measure the total flavonoid contents in the extract. Determination of total phenolic contents in the samples was determined by the Folin-Ciocalteu method [18].

#### 2.2.2. Untargeted Metabolomics Analysis

The extracts were filtered using a microporous filter membrane (0.22 μm pore size) prior to being stored in the injection vial for LC-MS/MS (UPLC, LC-30A, Shimadzu, Kyoto, JapanMS, TripleTOF6600+, SCIEX, Foster City, CA, USA) detection. The chromatographic parameters were set as follows: column: Waters ACQUITYUPLCHSST 31.8 µm, 2.1 mm × 100 mm; mobile phase A: ultrapure water (0.1% formic acid); mobile phase B: acetonitrile (0.1% formic acid); instrumental column temperature: 40 °C; flow rate: 0.40 mL/min; injection volume: 4 µL. The ESI source operating parameters were as follows: source temperature, 550 °C; ion spray voltage, 5500 V (positive ion mode)/−4500 V (negative ion mode). Peaks with >50% missing in each group of samples were filtered, and blanks were KNN-filled. The peak areas were then corrected using the SVR method. The corrected and filtered peaks were then identified by searching the Metware database (Wuhan Metware Biotechnology Co., Ltd., Wuhan, China) for metabolite identification.

Substances with a combined identification score of 0.5 or above and a CV value of less than 0.5 for QC samples were extracted and then combined in positive and negative modes, and those with the highest qualitative grade and the smallest CV value were retained.

#### 2.2.3. HPLC Conditions

High-performance liquid chromatography (HPLC) was performed to determine the content of hederagenin, tannic acid, and ferulic acid in the SMFW. The separation was achieved using an Xtimate C18 (Welch Materials (Shanghai) Co., Ltd., Shanghai, China) at 30 °C. The mobile phase composition for the analysis of hederagenin was solvent A (acetonitrile) and B (an aqueous solution of 0.02% phosphoric acid in water), with a wavelength of 205 nm. Elution was performed using a 35 min gradient: 0–2 min (10%A, 90%B), 2–25 min (80%A, 20%B), 25–30 min (80%A, 20%B), 30–20.1 min (10%A, 90%B), and 30.1–35 min (10%A, 90%B). The mobile phase for ferulic acid detection and tannic acid detection was composed of solvent A (methanol) and B (an aqueous solution of 0.01% formic acid). In both cases, the detection wavelength was 280 nm. Elution was performed using a 22 min gradient: 0–2 min (10%A, 90%B), 2–7 min (40%A, 60%B), 7–17 min (70%A, 30%B), 17–18 min (10%A, 90%B), and 18–22 min (10%A, 90%B). The flow rate was set at 1 mL/min, with an injection volume of 10 μL. The compounds were identified by comparing their retention times with those of authentic standards, and quantification was achieved using linear regression analysis.

### 2.3. In Vitro Antioxidant Assays

#### 2.3.1. DPPH Radical Scavenging Assay

The DPPH scavenging activity of samples was measured according to the method described by Chen et al., with modifications [19]. Briefly, DPPH (150 μmol/L) solution and extracts of different dilutions were 1:1 (*v*/*v*) mixed and incubated in darkness for 30 min followed by absorbance measurement at 517 nm. Vitamin C (Vc) was the positive control. The DPPH radical scavenging rate was calculated as follows:

DPPH scavenging rate (%) = [1 − (A_a_ − A_b_)/A_c_] × 100 (1)

A_a_ refers to the absorbance of the positive control or sample; A_b_ refers to the absorbance of replacing DPPH with ethanol; and A_c_ refers to the absorbance of distilled water.

#### 2.3.2. Superoxide Anion Radical Scavenging Assay

The superoxide anion radical (O_2_^−^) scavenging effect of extracts was measured according to the Sonu et al. method, with modifications [20]. Briefly, 5 mL Tris-HCl buffer (50 mM, pH 8.34) and 0.5 mL diluted extracts were mixed and incubated at room temperature for 20 min. Subsequently, 10 μL of pyrogallol (5 mM) was added and the obtained mixture was incubated at room temperature for 20 min. Vitamin C (Vc) was the positive control. The O_2_^−^ scavenging rate was calculated as follows:

O_2_^−^ scavenging rate (%) = [1 − (A_a_ − A_b_)/A_c_] × 100 (2)

A_a_ refers to the absorbance of the positive control or sample; A_b_ refers to the absorbance of replacing pyrogallol with water; and A_c_ refers to the absorbance of distilled water.

#### 2.3.3. ABTS Radical Scavenging Activity

The ABTS free radical scavenging assay was performed according to the method described by Liu et al., with modifications [21]. Briefly, 7.4 mM ABTS solution and 2.6 mM potassium persulfate solution were 1:1 (*v*/*v*) mixed and incubated at room temperature in the dark for 12~15 h to obtain the ABTS stock solution. Samples of 40 μL were mixed with 160 μL ABTS solution in a 96-well plate and incubated in the dark at room temperature for 6 min followed by absorbance measurement at 734 nm. Vc was used as the positive control. The ABTS scavenging rate was calculated as follows:

ABTS^+^ scavenging rate (%) = [1 − (A_a_ − A_b_)/A_c_] × 100 (3)

A_a_ refers to the absorbance of the positive control or sample; A_b_ refers to the absorbance of replacing ABTS solution with water; and A_c_ refers to the absorbance of ethanol.

#### 2.3.4. FRAP Assay

The ability of extracts to reduce Fe^3+^ to Fe^2+^ was tested using the FRAP assay [22]. Briefly, the 10 mM 2,4,6-tripyridyltriazine solution, 20 mM FeCl_3_ solution, and acetic acid buffer (pH 3.6) were mixed as a working solution in the volume ratio of 1:1:10. A total of 20 μL diluted extract was added to 180 μL of FRAP reagent. The obtained mixture was incubated at room temperature in the dark for 5 min, followed by absorbance measurement at 596 nm. Vc solution was used as a positive control. The concentration of FeSO4 in the solution after the reaction was calculated according to the standard curve.

### 2.4. Antibacterial Assay

*C. acnes* (purchased from China General Microbiological Culture Collection Center) suspension in logarithmic phase was centrifuged and resuspended in PBS to an OD value of 0.09 (bacterial concentration of 1 × 10^8^ CFU/mL). Then, 100 μL of the 100 times-diluted *C. acnes* solution was evenly spread on the RCM medium (J&K Scientific, Beijing, China) plate. The plates were punched with holes, in which 20 μL of extracts were added. PBS solution was the negative control. The plates were placed in an anaerobic environment in a 37 °C incubator for 48–72 h. The diameter of the transparent inhibition zone was measured by vernier calipers.

The minimum inhibitory concentration (MIC) of SMFW and SMPW was determined by the two-fold dilution method referring to Resmi’s [23] method, with modification. Columns 2–10 of the 96-well plates were filled with 100 μL of RCM liquid medium, and the first column was filled with 200 μL of SMFW and SMPW stock solution. Then, 100 μL was taken and added to column 2 and mixed well, and then sequentially added to column 10 for dilution. The concentrations of the microdilution plates were thus established at 100%, 50%, 25%, 12.5%, 6.25%, 3.125%, 1.5625%, 0.78125%, 0.390625%, and 0.1953125%, respectively. Subsequently, 100 μL of a 10^6^ CFU/mL bacterial suspension was added to each well. Concomitantly, the wells containing solely bacterial suspension were designated as the positive control, while the wells containing exclusively culture medium were designated as the negative control. After an incubation at 37 °C for 24 to 40 h, the clarity of the solution in the wells devoid of precipitation was considered to be the MIC value of the extract for the bacterium.

### 2.5. In Vitro Anti-Inflammatory Activity

#### 2.5.1. Cell Viability Assay

THP-1 cells with a density of 8 × 10^5^ cells/well in 96-well plate were kept in an incubator (37 °C, 5% CO_2_) overnight. PMA was added to cells with a final concentration of 100 ng/mL and cells were cultured for 48 h. Then, diluted extracts were added to each well and incubated for 24 h.

HaCaT cells were seeded into 96-well plates at a density of 1 × 10^4^ cells/well and incubated overnight (37 °C, 5% CO_2_). Subsequently, diluted extracts were added to each well followed by 24 h incubation under identical culture conditions.

Afterwards, the medium was removed and 10 µL of serum-free medium containing CCK8 solution was added to each well and incubated in an incubator for 60 min. The absorbance of each well was measured at 450 nm. The cell viability was calculated as follows:

Cell viability (%) = [(A_a_ − A_b_)/(A_c_ − A_b_)] × 100 (4)

A_a_ refers to the absorbance of sample; A_b_ refers to the absorbance of replacing serum-free medium containing CCK8 solution with serum-free medium; and A_c_ refers to the absorbance of replacing extracts with PBS.

#### 2.5.2. Cytokine Measurement

THP-1 cells were cultured in 6-well plates with a density of 1 × 10^6^ cells/mL and treated by 100 ng/mL of PMA for 48 h. Then, cells were isolated and washed twice with PBS, followed by the addition of LPS (1 µg/mL) and diluted extracts. After 6 h of incubation, the levels of pro-inflammatory cytokines in cell supernatant were determined by ELISA kits according to the manufacturer’s instructions. DEX (100 μg/mL) was used as the positive control.

### 2.6. RNA-Seq Analysis

THP-1 macrophages were collected 6 h after the extract treatment. Subsequently, 1 mL of Trizol Reagent (Life Technologies, Carlsbad, CA, USA) was added and mixed evenly followed by snap-freezing with liquid nitrogen. Three biological replicates were included in each group. The sequencing process was performed by BGI Genomics Co., Ltd. (Shenzhen, China). Gene expression quantification was performed using RSEM (v1.3.1) software. DESeq2 (v1.4.5) was used for differentially expressed gene analysis, with the criteria of |log_2_FC| ≥ 1, Q-value < 0.05.

### 2.7. Quantitative Real-Time PCR (qRT-PCR) Analysis

Total RNA was extracted using TRIzol methods and cDNA was synthesized using PrimeScript™ FAST RT reagent Kit with gDNA Eraser (TakaRa, Kusatsu Shiga, Japan). The qRT-PCR analysis was performed using the TB Green^®^ Premix Ex Taq™ II FAST qPCR (TakaRa, Japan) according to the manual. The qRT-PCR reaction conditions were as follows: 95 °C for 30 s, 40 cycles of 95 °C for 5 s, and 50–60 °C for 10 s. GAPDH was used as the reference gene and relative expression levels of target genes were calculated according to the 2^−△△Ct^ method. Primer sequences are listed in Supplementary Table S1.

### 2.8. Statistical Analysis

All measurements were performed on at least three replicates, and all values are expressed as mean ± standard deviation (SD). Statistical analysis was performed using Student’s *t*-test in GraphPad Prism 10.2.1 with *p* ≤ 0.05 as a statistically significant difference.

## 3. Results

### 3.1. Phytochemical Analysis of SMFW

#### 3.1.1. Total Saponin, Phenolic, and Flavonoid Contents

**Figure 1:**
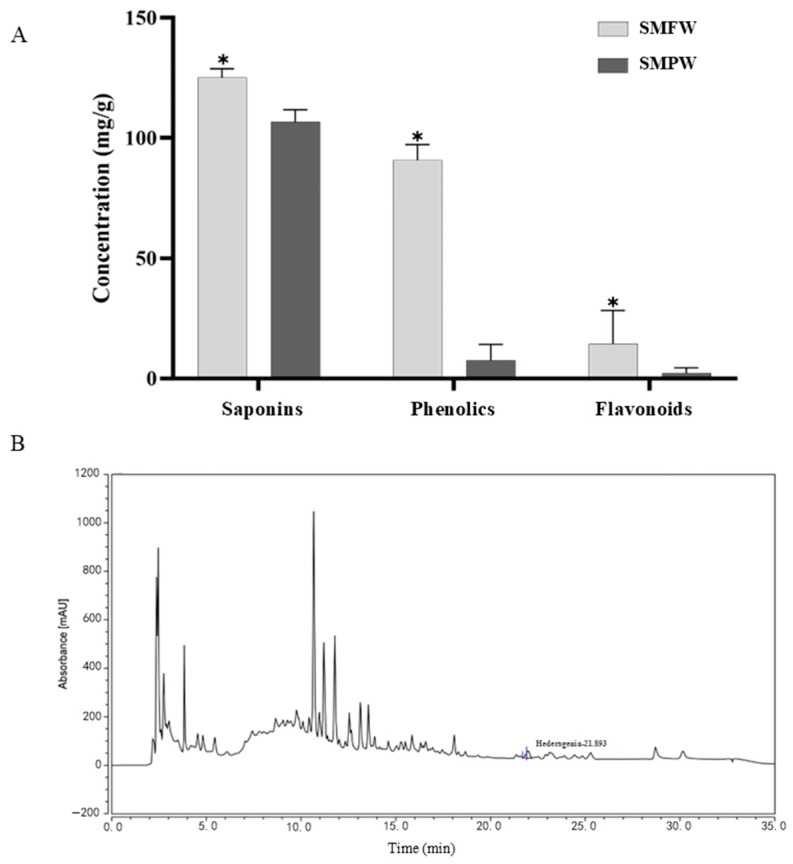
(**A**) Total saponin, phenolic, and flavonoid contents in SMFW and SMPW. SMFW represents *S. mukorossi* flower water extracts; SMPW represents *S. mukorossi* pericarps water extracts. (**B**) Hederagenin fractions from high-performance liquid chromatography. Each bar represents mean ± SD (*n* = 3). * represents a significant difference between SMFW and SMPW (*t*-test, *p* ≤ 0.05).

The pericarp of *S. mukorossi* has been extensively studied because of its high saponin contents. Therefore, the bioactive substance contents in SMFW and *S. mukorossi* pericarp water extracts (SMPW) were compared, as well as the subsequent in vitro bioactivities. The same extraction process was performed using *S. mukorossi* pericarp to obtain SMPW. The total saponin, phenolic, and flavonoid contents of SMFW were 128.8 ± 0.94 mg/g, 97.21 ± 1.87 mg/g, and 28.23 ± 0.60 mg/g, respectively, which were significantly higher than that of SMPW (Figure 1A).

#### 3.1.2. Untargeted Metabolomics Analysis of SMFW

In order to gain a comprehensive understanding of the composition of SMFW, the metabolite profile of SMFW was analyzed using LC-MS/MS. A total of 2422 metabolites were detected, and a detailed list of all identified compounds in SMFW is provided in Supplementary Table S2. Specifically, 87 phenolic acids, 121 flavonoids, and 94 terpenes were identified, including bioactive compounds such as hederacoside C, saponin D, ferulic acid, quercetin, quercetagitrin, and epigallocatechin, etc.

#### 3.1.3. HPLC Analysis

Previous phytochemical studies have identified hederagenin as a constituent in the pericarp extracts of *S. mukorossi* [24]. Extensive pharmacological investigations have demonstrated the anti-inflammatory [25], antibacterial [26], and antitumor [24] properties of hederagenin. In addition, it has been reported that *S. mukorossi* extracts are rich in various bioactive components such as ferulic acid and tannic acid [27,28]. Therefore, hederagenin, ferulic acid, and tannic acid were selected as target analytes for HPLC analysis. Table 1 provides the linear calibration curves for the standard solutions of the hederagenin. As shown in Figure 1B, the retention time for hederagenin was 21.893 min. The total amount of hederagenin in the SMFW was 23.2706 µg/mL. However, tannic acid and ferulic acid were not detected in SMFW, which may be attributable to the fact that their levels were below the detection limit.

### 3.2. Antioxidant Activity

**Figure 2:**
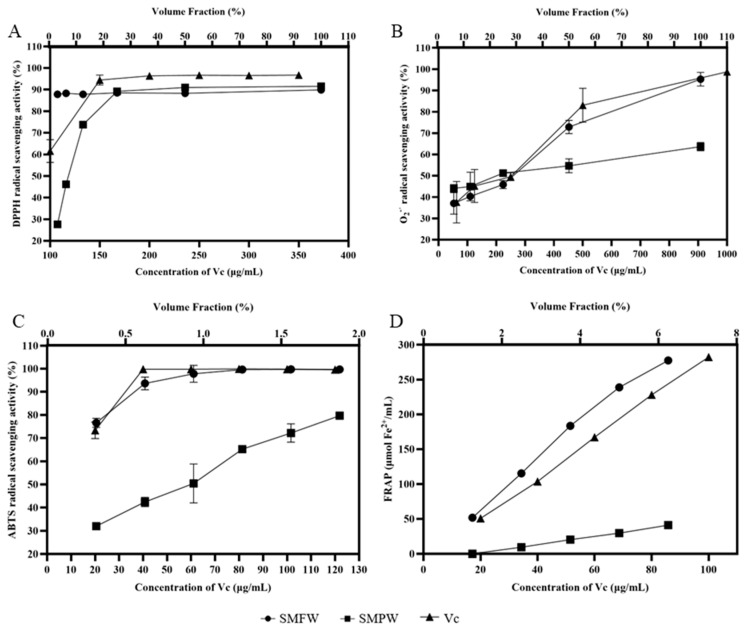
Antioxidant capacities of SMFW and SMPW measured by (**A**) DPPH assay, (**B**) O_2_^−^ assay, (**C**) ABTS assay, and (**D**) FRAP assay. Results are presented as mean ± SD (*n* = 3).

In Figure 2A, the DPPH^·^ scavenging rate of 3.125% SMFW was 88.25%, which was higher than that of 3.125% SMPW (28.05%) and 100 μg/mL of Vc (61.52%). The DPPH^·^ scavenging rates of SMFW and SMPW at a volume fraction of 100% were 90.28% and 91.98%, respectively. These results indicated that SMFW possessed strong DPPH^·^ scavenging activity. The O_2_^−^ scavenging rate was increasing with the increased concentration of SMFW and SMPW (Figure 2B). The O_2_^−^ scavenging rate of 100% SMFW was 95.15%, which was significantly higher than that of SMPW (63.54%) and comparable to 1000 μg/mL of Vc (98.77%). Similarly, SMFW exhibited higher ABTS^+^ scavenging activity than SMPW (Figure 2C). The ABTS^+^ scavenging rate of 1.88% SMFW was 99.54%, which was significantly higher than that of SMPW (79.47%) and equivalent to 120 μg/mL of Vc (99.63%). As shown in Figure 2D, the iron reduction capacity of 6.25% SMFW was equivalent to that of 100 μg/mL Vc. The reducing rate of 6.25% SMPW was lower than that of 20 μg/mL Vc solution and 1.25% SMFW. Therefore, the iron ion reduction capacity of SMFW is superior to that of SMPW.

### 3.3. Antibacterial Activity Against C. acnes

**Figure 3:**
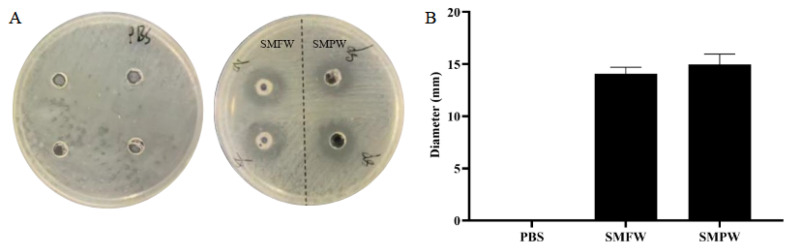
Antibacterial activity of SMFW and SMPW against *C. acnes*. (**A**) Growth of *C. acne* in the inhibition zone assay. (**B**) Quantification of inhibition zone diameter. Results are presented as mean ± SD (*n* = 3).

As shown in Figure 3A, SMFW and SMPW application resulted in distinct inhibition zones on *C. acnes*. The inhibition zone diameters of SMFW and SMPW were 14.08 ± 0.63 mm and 14.98 ± 0.98 mm, respectively (Figure 3B). The outcomes of MIC experiments demonstrated that both SMFW and SMPW were clarified without precipitation in the wells at column 7, and the precipitation was evident at column 8, thereby indicating their minimum inhibitory dose as 1.5625%.

To enable a more visual comparison of the differences in antioxidant and antimicrobial activities between SMFW and SMPW, we summarized the results in Table 2 using data from Figure 2 (antioxidant index) and Figure 3 (antimicrobial profile).

### 3.4. Anti-Inflammatory Activity

**Figure 4:**
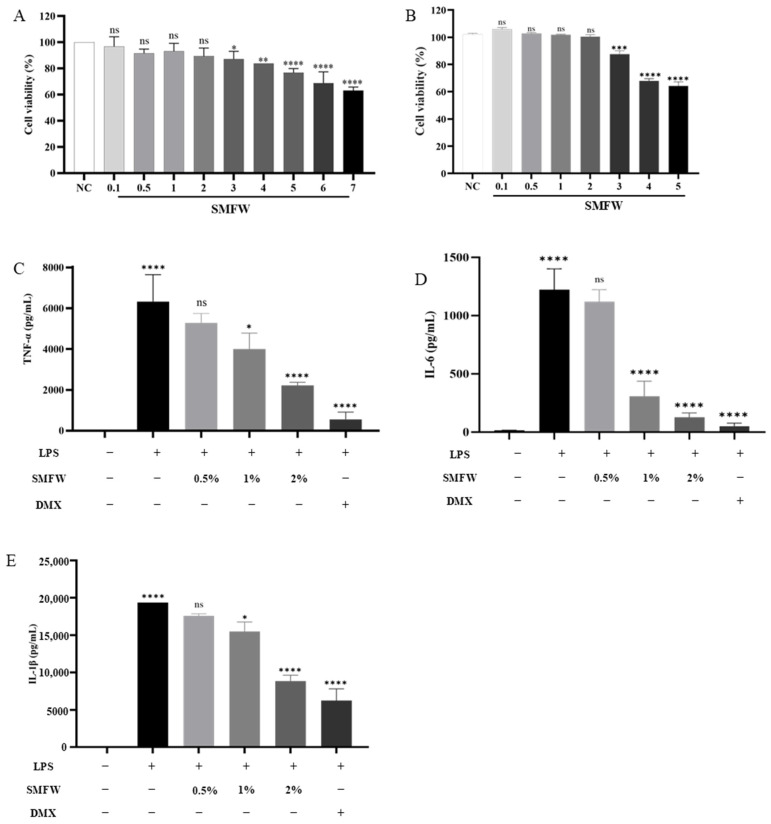
Anti-inflammatory activity of SMFW. Cytotoxicity of SMFW on THP-1 cells (**A**). Cytotoxicity of SMFW on HaCaT cells (**B**). Effects of SMFW on LPS-induced inflammatory factors in THP-1: (**C**) TNF-α, (**D**) IL-6, and (**E**) IL-1β. Results are presented as mean ± SD (*n* = 3). NC, negative control; LPS, lipopolysaccharide; DMX, dexamethasone; ns, no significance; * *p* < 0.05, ** *p* < 0.01, *** *p* < 0.001, **** *p* < 0.0001.

To explore the concentration that is safe to THP-1 cells, SMFW was tested for its cytotoxicity by CCK-8 assay. As is shown in Figure 4A, compared with the negative control (NC) of PBS, low concentrations of SMFW (0.1~2%) displayed no significant effects on the viability of THP-1 cells (*p* > 0.05). When the concentration exceeded 3%, the cell viability decreased significantly. Specifically, 5% of SMFW led to the viability rate of less than 80% (*p* < 0.0001). To investigate the effects of SMFW on cutaneous cells, the dose-dependent cytotoxic effects of SMFW on HaCaT were quantitatively evaluated using the CCK-8 cell viability assay (Figure 4B). The results demonstrated that SMFW at concentrations ranging from 0.1% to 2% showed no significant effects on the viability of HaCaT cell (*p* > 0.05). Therefore, 0.5%, 1%, and 2% of SMFW were selected for follow-up experiments.

The effects of SMFW on the content of TNF-α, IL-6, and IL-1β in LPS-induced THP-1 macrophages were evaluated (Figure 4C–E). The levels of TNF-α, IL-6, and IL-1β in the control group without LPS treatment were very low. Compared to the control group, the levels of TNF-α, IL-6, and IL-1β in the model group were significantly increased (*p* < 0.001), indicating that the inflammation cellular model was successfully established. Compared to the model group, SMFW treatment with concentrations of 1% and 2%, but not 0.5%, exhibited significant inhibitory effects on the secretion of three inflammatory cytokines. In addition, the inhibition activity increased in a concentration-dependent manner. These results indicated that SMFW possessed anti-inflammatory activity and can be used as an anti-inflammatory raw material.

### 3.5. Transcriptome Analysis

To further investigate the underlying mechanisms of SMFW’s anti-inflammatory activity, we performed RNA-seq analysis on three sample groups: Control (no LPS stimulation), Model (LPS stimulation), and SMFW (LPS stimulation and 2% SMFW treatment).

The clean reads ratio of each sample varied from 97.16% to 99.25%, indicating that the sequencing data quality was good for subsequent analysis (Supplementary Table S3). The correlation cluster diagram and PCA plot analysis revealed good repeatability of three replicates within the groups and large variations between different groups (Supplementary Figure S1). A total of 17,045 genes were detected and differentially expressed genes (DEGs) of Model vs. Control and SMFW vs. Model were screened with the criteria of |log_2_ FC| ≥ 1, Q-value < 0.05 and listed in Supplementary Tables S4 and S5.

#### 3.5.1. DEGs Between Model and Control

**Figure 5:**
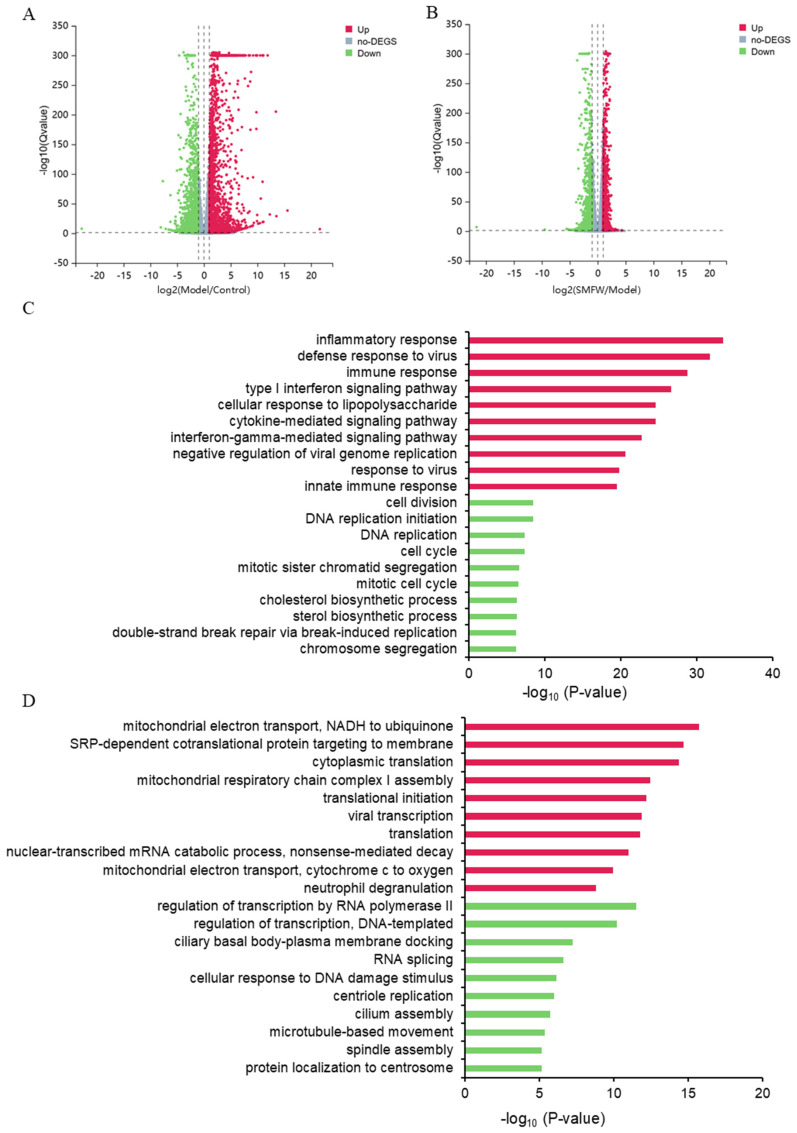
Differential analysis in mRNA expression levels between the Control group, Model group, and SMFW group. (**A**,**B**) Volcano plots showing the relative abundance of transcripts. The red and green dots indicate significantly up- and down-regulated genes, respectively (|log2 FC| ≥ 1, Q-value < 0.05). The gray dots are non-significantly different genes. (**C**,**D**) GO enrichment analysis of DEGs in Model vs. Control and SMFW vs. Model. Results of up- and down-regulated genes are presented as red and green, respectively.

Compared to Control group, a total of 4317 genes were differentially expressed in Model group, in which 2059 genes were up-regulated and 2258 genes were down-regulated (Figure 5A). In Figure 5C, the top 10 significantly enriched GO terms in biological process category of up-/down-regulated DEGs are shown. For the up-regulated DEGs, enriched GO terms included inflammatory response, defense response to virus, immune response, type I interferon signaling pathway, cellular response to lipopolysaccharide and cytokine-mediated signaling pathway, etc. These enriched terms demonstrated inflammatory responses induced by LPS in THP-1 cells. For the down-regulated DEGs, enriched GO terms included cell division, DNA replication initiation, DNA replication, cell cycle, etc. These enriched terms suggested the cell cycle and cell division were impeded in LPS-treated cells.

#### 3.5.2. DEGs Between SMFW and Model

Comparison of SMFW and Model group identified a total of 1147 up-regulated and 1427 down-regulated DEGs (Figure 5B). The top 10 significantly enriched GO terms in the biological process category of up-/down-regulated DEGs are shown in Figure 5D. For the up-regulated DEGs, the enriched GO terms included mitochondrial electron transport and NADH to ubiquinone, SRP-dependent co-translational protein targeting to membrane, cytoplasmic translation, mitochondrial respiratory chain complex I assembly, translational initiation, etc. For the down-regulated DEGs, the enriched GO terms included regulation of transcription by RNA polymerase II, regulation of transcription (DNA-templated), ciliary basal body-plasma membrane docking, RNA splicing, cellular response to DNA damage stimulus, etc. These results suggested SMFW treatment possibly impacted the mitochondrial function and down-regulated transcription processes.

#### 3.5.3. DEGs with Inverse Trends in Model vs. Control and SMFW vs. Model

**Figure 6:**
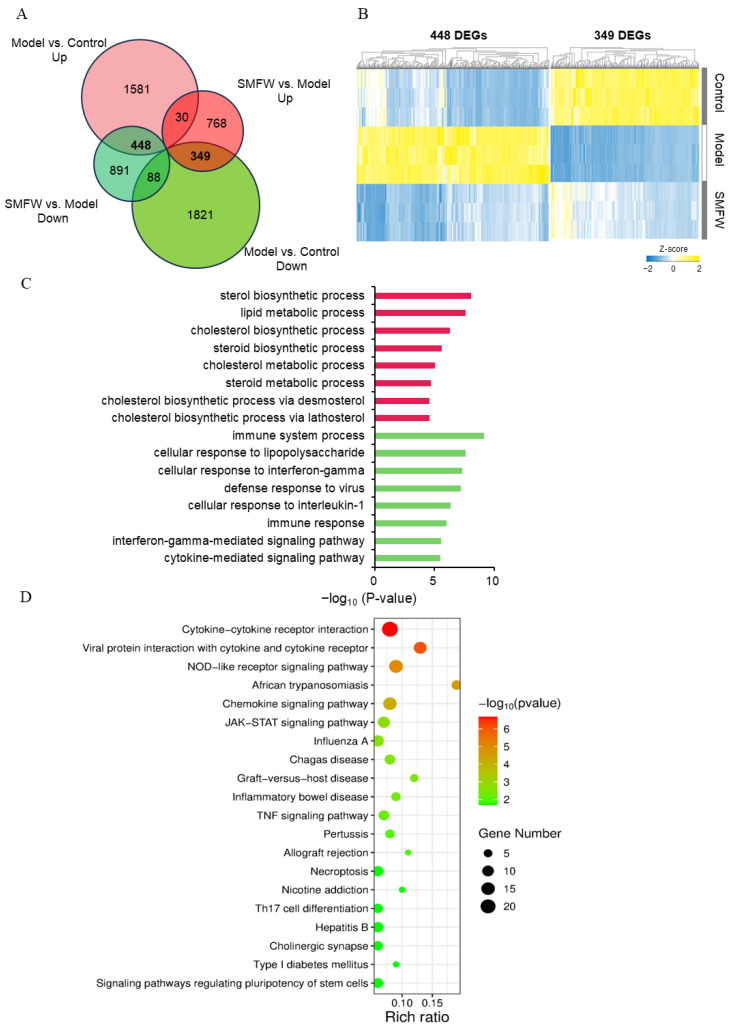
Analysis of SMFW-reversed DEGs. (**A**) Venn diagram showing the overlap between Model vs. Control DEGs and SMFW vs. Model DEGs. (**B**) Hierarchical clustering and heatmap of SMFW-reversed DEGs. The 448 DEGs are up-regulated in Model vs. Control, but down-regulated in SMFW vs. Model; the 349 DEGs are down-regulated in Model vs. Control, but up-regulated in SMFW vs. Model. The yellow and blue color indicate relatively high and low expression levels, respectively. (**C**) GO enrichment analysis for 349 DEGs (red) and 448 DEGs (green). (**D**) KEGG enrichment analysis for 448 DEGs.

To further explore the mechanism by which SMFW exerts its anti-inflammatory effect, the overlap of DEGs in the two contrasts Model vs. Control and SMFW vs. Model was analyzed by Venn diagram (Figure 6A). A total of 448 DEGs were up-regulated in Model vs. Control, but down-regulated in SMFW vs. Model, and 349 DEGs were down-regulated in Model vs. Control, but up-regulated in SMFW vs. Model. We focus on these two groups of DEGs with reversed expression patterns in two comparisons. The clustering heatmap represented the expression characteristics of these two groups of DEGs (Figure 6B). Compared to Control group, expression levels of 448 DEGs were highly induced in the Model group, while with SMFW treatment, their expression levels were significantly decreased and were similar to the Control level. However, the 349 DEGs that were down-regulated in Model group were still expressed at relatively low levels after SMFW treatment compared to Control group.

As is shown in Figure 6C, the enriched GO terms in the biological process category of 349 DEGs were sterol biosynthetic process, lipid metabolic process, cholesterol biosynthetic process, steroid biosynthetic process, etc. The enriched GO terms of 448 DEGs were immune system process, cellular response to lipopolysaccharide, cellular response to interferon-gamma, defense response to virus, etc.

**Figure 7:**
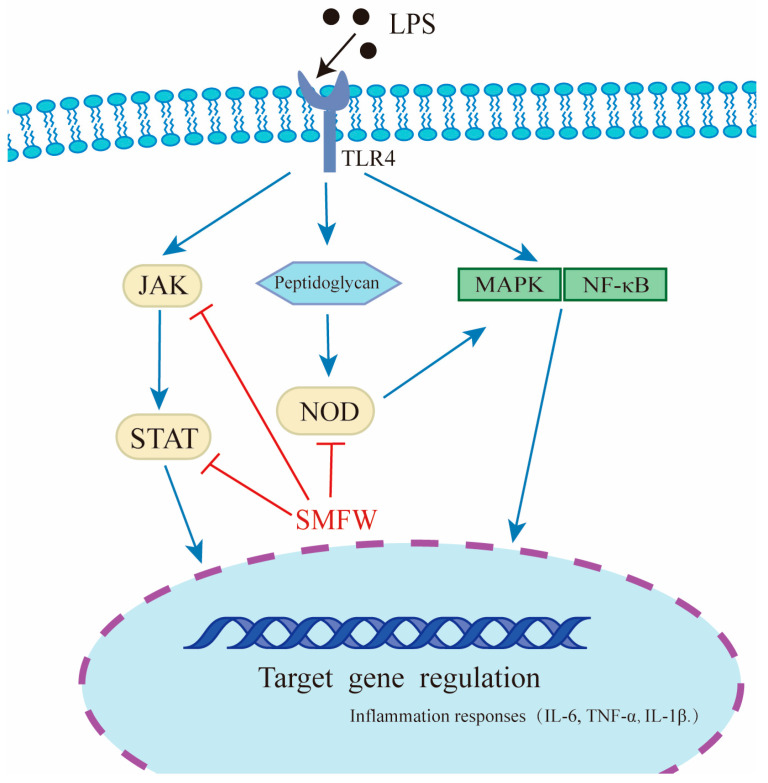
Proposed molecular mechanism of SMFW in anti-inflammatory regulation: a schematic overview.

We performed KEGG pathway enrichment analysis on the 488 DEGs and presented the top 20 enriched pathways (Figure 6D). Pathways including cytokine-cytokine receptor interaction, viral protein interaction with cytokine and cytokine receptor, NOD-like receptor signaling pathway, JAK-STAT signaling pathway, chemokine signaling pathway, TNF signaling pathway, etc., were significantly enriched. The DEGs mapped to NOD-like receptor pathway included NOD2, MCP-1, IL-6, CASP5, etc., and the DEGs mapped to JAK-STAT pathway included JAK, STAT, SOCS (Supplementary Figure S2). Based on the RNA-seq data, we proposed that SMFW might exert anti-inflammatory effects by down-regulating the expression of key genes in JAK-STAT and NOD signaling pathways (Figure 7).

#### 3.5.4. Validation of Gene Expression by qRT-PCR

**Figure 8:**
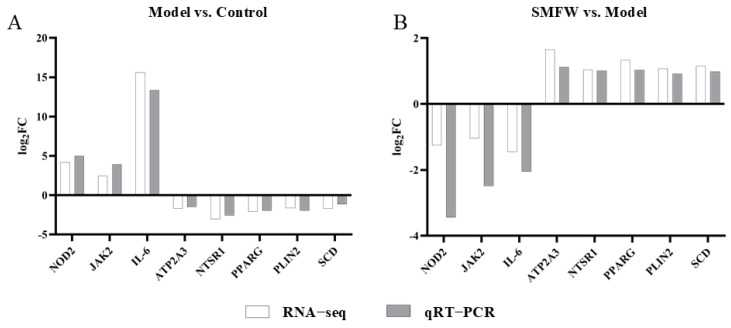
qRT-PCR validation of the RNA-sequencing results (**A**) Model vs. Control, (**B**) SMFW vs Model.

To validate the RNA-seq results, three genes from the 488 DEGs group (IL-6, NOD2, and JAK2) and five genes from the 349 DEGs group (ATP2A3, NTSR1, PPARG, PLIN2, and SCD) were selected for expression analysis by qRT-PCR (Figure 8). The expression trends of these genes were consistent in RNA-seq and qRT-PCR analysis, supporting the accuracy of the RNA-seq data.

## 4. Discussions

*S. mukorossi* extract is gaining popularity as a potent ingredient in various fields due to its high concentration of saponins, which possesses antioxidant and antibacterial properties [3]. The objective of this study was to evaluate the potential of SMFW in the treatment of acne, and to determine the underlying mechanisms of its anti-inflammatory activity, with a view to further utilize *S. mukorossi* resources.

Considering the safety and environment pollution risk of organic solvents, as well as the cost and energy consumption for solvent recovery, water was selected as the extraction solvent in this study. The higher level of total flavonoid content in SMFW compared to SMPW in our study is consistent with Xu et al.’s report [4]. Xu et al. compared the saponin and flavonoid contents in different organs of *S. mukorossi* (leaves, flowers, branches, roots, pericarp, seed coat, and seed kernel) and found that the pericarps contained the highest level of saponins, followed by flowers, whereas the leaves contained the highest level of total flavonoids, followed by the flowers, and total flavonoid content in the pericarps was relatively low. However, our results showed that the saponin content of SMFW was higher than that of SMPW, which might be because the extraction condition for SMPW was not optimized, resulting in a low extraction efficiency.

The non-targeted metabolomic analysis further revealed that SMFW are abundant in bioactive components such as phenolic acids, flavonoids, and terpenes. Phenolic acid, a secondary plant metabolite, has been shown to possess substantial antioxidant properties through its capacity to scavenge free radicals and enhance the activities of detoxification enzymes (e.g., those implicated in ROS/RNS metabolism) [29]. Flavonoids have been shown to possess antioxidant, anti-inflammatory, and anticancer properties, and their functional hydroxyl groups have the capacity to scavenge free radicals, as well as chelate metal ions in order to inhibit the generation of ROS [30]. Terpenoids are natural hydrocarbons that are produced by a wide range of plants, and play an important role in their antibacterial, antifungal, antiviral, anti-inflammatory, and antioxidant properties [31]. The results of our HPLC analysis demonstrated the presence of hederagenin in SMFW. Hederagenin has been shown to regulate the NF-*κ*B signaling pathway by inhibiting the activation of IKK*β*, thereby significantly reducing the production of inflammatory factors such as IL-6, IFN-*γ*, and TNF-*α* [32]. Furthermore, previous study demonstrated the efficacy of hederagenin in reducing the activity of p38 in a model of ethanol-induced liver injury [33]. The activation of p38 has been demonstrated to promote the production of inflammatory factors, including TNF-α, IFN-*γ*, TNF-*α*, and IL-6, by phosphorylating and activating transcription factors (NF-κB, AP-1, and ATF2) [34]. It was hypothesized that the anti-inflammatory activity of SMFW might be predominantly ascribed to hederagenin.

The development of acne has been demonstrated to be inextricably linked to the presence of ROS (reactive oxygen species) [35]. An accumulation of ROS can induce oxidative stress within the cells, resulting in cellular damage and an inflammatory response [36]. Thus, application of plant extracts with antioxidant activity is a prospective strategy for anti-acne treatment [37]. DPPH, O_2_^−^, and ABTS^+^ radical scavenging assays are commonly performed to assess antioxidant capacity of plant extracts in vitro [38,39], while FRAP is a classic method for evaluating the iron ion-reducing ability of plant extracts [40]. In our study, these four in vitro antioxidant assays revealed stronger antioxidant activities of SMFW compared to SMPW. Generally, phenolic compounds possess strong free radical scavenging abilities by providing hydrogen atoms to break the free radical chain [41]. Previous reports on the water, methanol, and ethanol extracts of *S. mukorossi* leaves and fruits revealed a strong correlation between the antioxidant activity and total phenolic content of different extracts [42]. Shah et al. [7] evaluated the antioxidant activity of methanol extract of *S. mukorossi* stem bark and its fractions. They found antioxidant assays including DPPH· scavenging activity, iron chelating assay, and reducing power assay showed positive and significant correlations with the total phenolic and flavonoid contents. Thus, the stronger antioxidant activities of SMFW compared to SMPW may result from the higher content of total phenolics and flavonoids in SMFW.

*C. acnes* is an anaerobic pathogen that can induce localized inflammatory responses in acne development [43]. Consequently, the inhibition of the proliferation of *C. acnes* in the skin has been identified as a therapeutic strategy for the treatment of acne. Our results demonstrated that both SMFW and SMPW could inhibit the proliferation of *C. acnes*. Saponins are the major constituents of *S. mukorossi.* A saponin fraction isolated from the *S. mukorossi* extract demonstrated strong antibacterial activity against *C. acnes* [44]. Wei et al. found that *S. mukorossi* saponins possess the capacity to impede bacterial proliferation by modifying the fatty acid composition of *C. acnes* and compromising its cell membrane integrity [45]. Mukurozisaponin E1, Rarasaponin II, Mukurozisaponin G, and Rarasaponin VI were found to contribute to these biological activities. The saponin fraction purified from the fermentation liquid-based water extract (SWF) showed the best antibacterial activity against *C. acnes* with a MIC of 0.06 mg/mL, which was 33-fold of its parent SWF (with a MIC of 2.0 mg/mL) [6]. The observed antibacterial activity of SMFW and SMPW against *C. acnes* might be due to the presence of the antimicrobial saponins, but the specific components remain to be elucidated.

LPS treatment is a well-established in vitro inflammation model, which involves the induction of pro-inflammatory cytokine secretion in THP-1 cells. Among the various cytokines secreted, TNF-α, IL-6, and IL-1β have been identified as particularly significant indicators of inflammation; overexpression of this pro-inflammatory factor will generate internal immune imbalance and trigger inflammatory cascade, resulting in widespread pathological injuries [46]. In the present study, SMFW treatment significantly reduced the secretion of IL-6, TNF-α, and IL-1β in cells when compared with the model group, suggesting an anti-inflammatory activity of SMFW. Previous studies reported that the stem bark of *S. mukorossi* exhibited a therapeutic potential for inflammatory disorders [7]. The oral administration of fractions of *S. mukorossi* stem bark methanol extract was found to inhibit edema at a dose of 300 mg/kg in the carrageenin-induced paw edema assay in Sprague-Dawley rats. The active substance responsible for the anti-inflammatory activity might be the triterpene saponin constituents. Analysis including constituent isolation and structural analysis are needed for further verification.

The mechanisms of the anti-inflammatory effect of *S. mukorossi* extract were further explored by RNA-seq technology. GO enrichment analysis showed that cell cycle and cell division were impeded in Model group compared to Control group. This result is consistent with previous reports that LPS treatment induced G0/G1 cell cycle arrest in THP-1 cells [47,48]. Overall, comparison of Model and Control proved that the LPS treatment stimulated inflammation successfully. Importantly, we found that 448 DEGs were up-regulated in Model vs. Control, but down-regulated in SMFW vs. Model. KEGG pathway analyses showed that the 448 DEGs were predominantly enriched in cytokine–cytokine receptor interactions, NOD-like receptor signaling pathway, and JAK-STAT signaling pathway, etc. Cytokines are soluble extracellular proteins or glycoproteins that can bind to specific receptors on the target cell surface. They are involved in immune responses, cell growth, differentiation, cell death, etc. [49]. The NOD-like receptor (NLR) signaling pathway is an important immune defense mechanism in organisms [50]. Nucleotide-binding oligomerization domain 2 (NOD2) is a typical NLR that can recognize muramyl dipeptide (MDP) in the cell membrane to activate downstream inflammatory mediators and pro-inflammatory factors through the NF-κB pathway [51]. A previous study reported that N-trans-p-coumaroyltyrosine, an amino acid amide isolated from *Abri Mollis Herba*, can inhibit LPS-induced pro-inflammatory cytokine (IL-6 and IL-1β) secretion and acute inflammatory injury in zebrafish, and the underlying mechanism may be through key genes in the NOD-like receptor signaling pathway [52]. Our results showed that SMFW treatment significantly decreased the expression of NOD2 compared to Model group, suggesting that SMFW might exert anti-inflammatory effects through the NOD-like receptor signaling pathway. In addition, the JAK-STAT signaling pathway is also implicated in the pathogenesis of acne [53]. In the JAK2/STAT3 signaling pathway, translocation of phosphorylated STAT3 dimers to the nucleus enhances the transcription and expression of target genes related to inflammatory factors such as IL-6. Wang et al. found that protosappanin A, isolated from *Caesalpinia sappan* L., exerted an anti-neuroinflammation effect by significant down-regulation of the phosphorylation of JAK2 and its downstream signaling mediator STAT3 in a dose-dependent manner in LPS-stimulated murine BV2 microglia [54]

Collectively, it can be hypothesized that SMFW may also alleviate inflammation induced by LPS via the JAK-STAT pathway.

## 5. Conclusions

This study reported that the contents of total phenols and total flavonoids in SMFW were higher than those in SMPW. SMFW exhibited antioxidant, antibacterial, and anti-inflammatory properties in in vitro experiments. RNA-seq analysis indicated that SMFW may alleviate inflammation by regulating the NOD-like receptor and JAK-STAT signaling pathways. In summary, SMFW has shown potential for anti-acne efficacy, and can be used as a natural raw material in cosmetics.

It should be noted that, while the current experimental framework intentionally focused on anti-acne potential of SMFW at the cellular/molecular level, systematic in vivo verification including animal efficacy models and clinical trials should be conducted in the next research phase. Comparative studies with commercially available anti-acne products will enhance the translational value of these findings.

## Figures and Tables

**Table 1:** Linearity of the standard curves of hederagenin.

Compound	Calibration Equation ^a^	Retention Time (t_r_)	Correlation Coefficient (r^2^)
Hederagenin	Y = 0.0644x + 0.0306	21.893 min	0.9999

^a^ The variable X is the concentration of the standard (μg/mL), and the variable Y is the peak area.

**Table 2:** Comparison of antioxidant and antibacterial capacities of SMFW and SMPW.

In Vitro Activity		Volume Fraction	SMFW	SMPW
Antioxidant activity	DPPH scavenging rates (%)	6.25%	88.72 ± 0.66	46.53 ± 0.96
	O_2_^−^ scavenging rate (%)	6.25%	36.94 ± 3.73	44.01 ± 3.15
	ABTS scavenging rate (%)	1.25%	99.52 ± 0.23	65.05 ± 1.4
	FRAP value	1.25%	52.11 ± 0.96	0.43 ± 0.35
Antibacterial assay	Inhibition diameter sizes (mm)	100%	14.08 ± 0.63	14.98 ± 0.98

## Data Availability

Data will be made available on request.

## Supplementary Materials

The following supporting information can be downloaded at: https://www.mdpi.com/article/10.3390/cimb47050316/s1.

## Abbreviations

SMFW	*S. mukorossi* flower water extract
SMPW	*S. mukorossi* pericarp water extract
LPS	Lipopolysaccharide
DMX	Dexamethasone
DEG	Differential expressed genes
*S. mukorossi*	*Sapindus mukorossi* Gaertn
