# Supplementary material for: Evaluation of Sapindus mukorossi Gaertn Flower Water Extract on In Vitro Anti-Acne Activity
Source: Curr Issues Mol Biol. 2025 Apr 28;47(5):316. doi: 10.3390/cimb47050316 (PMC12110581; doi:10.3390/cimb47050316)
Supplement: Supplementary file 1 [file cimb-47-00316-s001.zip › FigureS1 and S2.pdf]

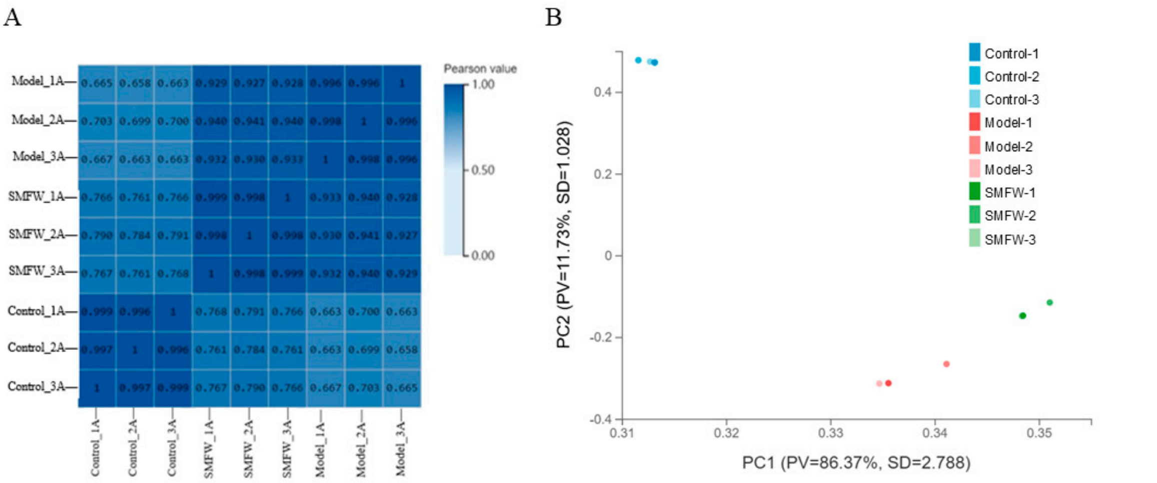

Figure S1. (A) Pearson correlation analysis and (B) PCA plot of samples from three experimental groups.

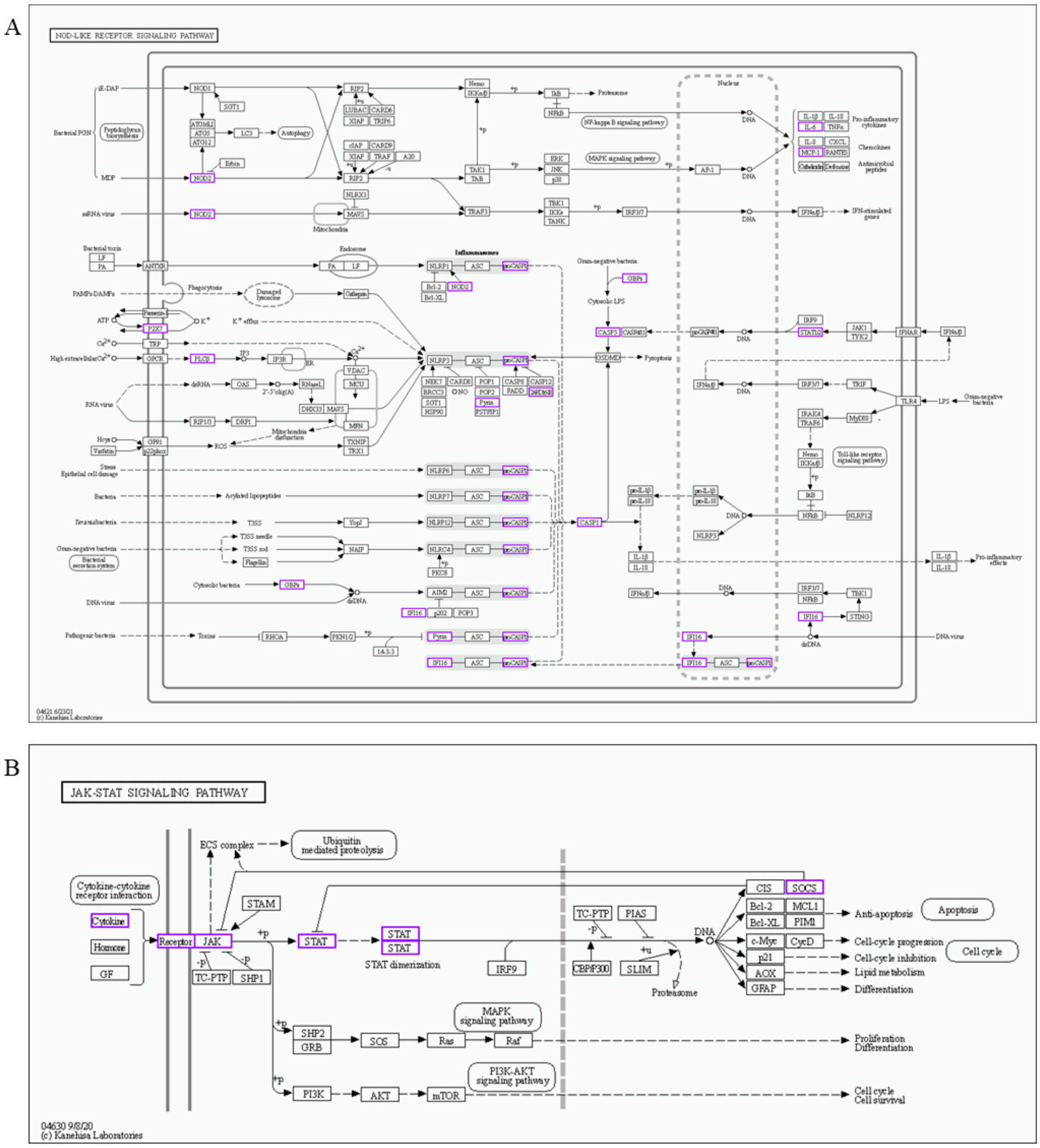

Figure S2. (A)NOD-like receptor signaling pathway and (B) JAK-STAT signalingpathway showing integrated transeriptome data.
